# Maternal ancestry and population history from whole mitochondrial genomes

**DOI:** 10.1186/s13323-015-0022-2

**Published:** 2015-03-10

**Authors:** Toomas Kivisild

**Affiliations:** Division of Biological Anthropology, University of Cambridge, CB2 1QH Cambridge, UK

**Keywords:** Maternal ancestry, MtDNA, Population history

## Abstract

MtDNA has been a widely used tool in human evolutionary and population genetic studies over the past three decades. Its maternal inheritance and lack of recombination have offered the opportunity to explore genealogical relationships among individuals and to study the frequency differences of matrilineal clades among human populations at continental and regional scales. The whole mtDNA genome sequencing delivers molecular resolution that is sufficient to distinguish patterns that have arisen over thousands of years. However, mutation rate is highly variable among the functional and non-coding domains of mtDNA which makes it challenging to obtain accurate split dates of the mitochondrial clades. Due to the shallow coalescent time of mitochondrial TMRCA at approximately 100 to 200 thousand years (ky), mtDNA data have only limited power to inform us about the more distant past and the early stages of human evolutionary history. The variation shared by mitochondrial genomes of individuals drawn from different continents outside Africa has been used to illuminate the details of the colonization process of the Old World, whereas regional patterns of variation have been at the focus of studies addressing questions of a more recent time scale. In the era of whole nuclear genome sequencing, mitochondrial genomes are continuing to be informative as a unique tool for the assessment of female-specific aspects of the demographic history of human populations.

## Review

### Introduction

Maternal inheritance [1], fast mutation rate [2], high copy number per cell [3,4], and the lack of recombination [5,6] were the features that brought mtDNA at the focus of evolutionary genetic studies in the 1980’s and 1990’s when the human genome sequencing had not been completed yet and the idea of whole nuclear genome level population genetics was only a daydream for population geneticists. The presence of mitochondria as energy producing small bacteria-like ‘power cells’ within our cells is one of the defining features of eukaryotes. The adoption of this organelle was a critical step in the earliest stages of our evolutionary history that allowed the cells of our ancestors to diversify in size and shape and to develop their characteristic feeding mode of a phagotrophic predator [7]. The special relationship between the hosting cell and the mitochondria also determines the specific aspects of the replication, transmission and population genetics of the DNA molecules in mitochondria, the variation of the mtDNA copy number by cell types and developmental stages and the small size and high gene density of mitochondrial genome (for review see [8]).

Humans along with western chimpanzees and eastern gorillas have remarkably low genetic diversity compared to other great apes [9]. Low genetic diversity means that for any nuclear gene one needs to sequence thousands or tens of thousands of base pairs to have a chance of finding SNPs that are informative for population genetic purposes. In the era of PCR and Sanger sequencing the high mutation rate made it more cost effective to uncover DNA sequence variation at the population scale from mtDNA than from any nuclear locus. Furthermore, the lack of recombination allowed the data from coding and non-coding regions of mtDNA to be combined into the shape of a phylogenetic tree. The branches of this ever-growing tree, as more data became available, could be labelled by distinctive restriction fragment length polymorphisms (RFLPs). As a result, the most common branches were assigned alphabetic labels that became to be known as mtDNA haplogroups [10].

The nomenclature of mtDNA haplogroups was introduced in the mid-1990s with A-G labels assigned to variation observed in Asian and American lineages [10,11], H-K to Europe [12] whereas only a single letter, L, was assigned to describe the highest level of variation observed in Africa in a study using an Asian outgroup [13]. The mtDNA nomenclature that is currently used (http://www.phylotree.org/) has a robust branch structure that has been determined through the rigorous and detailed analyses of the whole mtDNA genomes [14]. These topological details of the mtDNA phylogeny have been revealed step by step over the last two decades thanks to the contributions of many groups in covering with data ever increasing numbers of populations across the world and thanks to the advances in technology that eventually have led to the use of whole mtDNA sequencing as a routine approach in the field.

Robust inference of the phylogenetic tree and its high resolution has been important for various reasons. The initial RFLP based studies, for example, with limited number of polymorphic sites that were known in the early 1980’s had concluded that the root of human mtDNA was in Asia [15]. However, more comprehensive analyses of 195 polymorphic RFLP sites across the whole mtDNA sequence determined in 145 human placentas and two cell lines drawn from five geographically distinct populations [16] suggested that all variants observed in present day populations can be inferred to derive from a single female ancestor who was postulated to have lived approximately 200,000 years ago in Africa. However, these early phylogenies were not sufficiently robust, so that critics were able to produce alternative root topologies and African origins were repeatedly challenged and reclaimed in the following decade [17-20]. Although the RFLP studies and HVS-I sequencing based work often ended up showing high level of phylogenetic uncertainty they were the approaches taken at the time that provided the first insights into the mtDNA variation at continental scales. These efforts led to the formulation of research hypotheses that became actively debated and subject to further scrutiny, including, for example, the earliest attempts to define the genetic source and number of founding lineages of Native Americans [21] and of Polynesians [22,23], and relative contributions of Palaeolithic, Mesolithic and Neolithic gene flow in the peopling of Europe [24].

### Mutation rates and TMRCA of mtDNA variation

All evolutionary genetic studies that associate the patterns of mtDNA variation observed in human populations with time explicit models make assumptions about the molecular clock. The mutation rate of mtDNA in animals is known to be higher by at least an order of magnitude than the mutation rate in nuclear genes [2]. In vertebrates the mitochondrial mutation rate, in fact, is × 25 higher than nuclear DNA mutation rate whereas the opposite is true for most of the plants whose mitochondria evolve approximately × 20 slower than their nuclear genes [25]. However, the rates at which mutations occur or get fixed in mitochondria are not uniformly high along the molecule and its functional domains. The rate variation among sites and time dependence of substitution rates at the intra- and interspecies scales [26-29], along with issues related to germ line and somatic heteroplasmy [30] have been major challenges for getting accurate estimates of human mtDNA mutation rate. Heteroplasmy refers to the existence of different types of mtDNA in the same individual. Because of high copy number in most human tissues the levels of mtDNA heteroplasmy may vary from very low, <5%, that can be detected and studied now with the next generation sequencing methods (reviewed in [31]), to those up to 1:1 ratio. Most heteroplasmies are resolved within a few generations by the severe germ-line bottlenecks leading to the loss of many de novo mutations, an effect that needs to be considered when calibrating mutation rates from pedigree data [30]. Somatic heteroplasmies do not contribute to mutation rate and only a small fraction of germ-line mutations get fixed in genealogies. Further complicating factors include the directionality of the mutations [32] – most hypervariable positions are unstable only in the G- > A, T- > C direction (according to the L-strand convention of the reference sequence) and the 60 fold or higher effective transition/transversion rate biases [33].

Mechanisms emphasizing the damage exposure of one of the strands of the mtDNA molecule during the replication and/or transcription processes have been put forward to explain the high mutation rate of mtDNA, being both transition biased and strand specific [32,34,35]. Damage patterns that are caused by the deamination of the heavy strand lead to the excess of A to G and C to T transitions. Notably, transition hotspot patterns observed in aDNA are similar to those observed to be hypervariable in living populations suggesting that the underlying mechanism as how mutations accumulate in germ line is similar to the build-up of post-mortem damage [36].

The first estimates of mutation rate of the whole mtDNA that were used for the estimation of the TMRCA age were based on the divergence estimates of humans from the chimpanzee outgroup [37,38]. The apparent problem with this phylogenetic approach that used a distant outgroup for calibration of the mtDNA mutation rate was that it produced estimates which were at odds with the mutation rates estimated from pedigree data. In case of the hypervariable regions of the D-loop, several pedigree studies [39-42] had inferred mutation rates that were up to an order of magnitude higher than the phylogenetic rate [43] (Table 1). More recent studies using high coverage mtDNA sequence data suggest that these differences are mainly due to the detection of heteroplasmic states of somatic mutations which never get fixed in the germ lines [30]. Although it is encouraging to see recent aDNA based studies yielding concordant mutation rates for the whole mtDNA genome, substantial differences are still noted among functional domains of the molecule (Table 1).

**Table 1:** **Pedigree, phylogeny and aDNA-based estimates of mtDNA mutation rates (per bp per year × 10** ^**−8**^ **)**

**Domain**	**mtDNA**	**coding**	**RNA**	**PC1 + 2**	**PC3**	**syn**	**HVS-I**	**HVS-II**	**D-loop**
No sites	16569	15447	4021	6034	3017	4170	360	316	1122
**Pedigree data**									
Howell *et al*. 2003 [40]									47
Sigurdardottir *et al*. 2000 [42]							21		
Heyer *et al*. 2001 [39]							39		
Santos *et al*. 2005 [41]							7.4	43.3	17
Rebolledo *et al*. 2014 [30]	1.3								7.7
**Phylogenetic**									
Vigilant *et al*. 1991 [43]									5.7
Horai et al. 1995 [37]						3.9			
Ingman *et al*. 2000 [38]		1.7							
Tang *et al*. 2002 [44]		1							
Mishmar *et al*. 2003 [45]		1.26							
Kivisild *et al*. 2006 [26]						3.41			
Loogvali *et al*. 2009 [27]						3.00			
Soares *et al*. 2009 [28]	1.67	1.71	0.77	0.89	1.93		16.4	22.9	12.3
**Phylogeographic**									
Forster *et al*. 1996 [21]							18		
Ho and Endicott 2008 [46]		2.04							
Poznik *et al*. 2013 [47]	2.3								
**Ancient DNA**									
Fu *et al*. 2013 [48]	2.67	1.57		0.82	3.27				
Brotherton *et al*. 2013 [49]	2.4								
Fu *et al*. 2014 [50]	2.53								
Rieux *et al*. 2014 [51]	2.14		1.01	0.76	3.32		31.4		

‘PC1 + 2’, second and third codon positions of protein coding genes; PC3, third codon positions; syn, total number of PC1 and PC3 positions at which transitions will not lead to amino acid replacement which is equal to the sum over all fourfold (2026) and twofold (2144) degenerate sites.

Overall, the mutation rate of human mtDNA is over an order of magnitude higher than nuclear rate mainly because of the deamination based high transition rates which are >60 times higher than the transition rate in nuclear genome while the transversion rates are more similar, with only approximately × 5 higher rate than in nuclear genes. To put these rate estimates further into perspective, it is interesting to note that the per-generation mutation rate of mtDNA in humans, approximately 6 × 10^−7^, is approximately × 10 faster than that of Drosophila [52] while the per year mutation rate is × 100 slower because the generation time in Drosophila is just 10 days.

One of the questions addressed in mtDNA studies on global scale has been the age of the diversity in the locus. Different studies have yielded mtDNA TMRCA age estimates that are young relative to autosomal data and vary (depending on the dating technique and mutation rate being use) in the range of 100 to 200 thousand years ago (kya) [26,37,38,53-55]. These estimates are generally similar [47,56] to those based on Y chromosome or slightly younger [57] when considering the rare Y chromosome haplogroup A00 lineages that were recently found restricted to West Africans. The upper end of these time estimates falls to a period in the African fossil record that is associated with the first appearance of anatomically modern humans [58]. Considering that the time back to TMRCA of a genetic locus is determined primarily by the long term effective population size of the species, the age of TMRCA does not necessarily inform us about a biologically significant event, such as the origin of the species, unless the species went through a speciation bottleneck and was founded from a very small number of individuals. Genetic and fossil evidence for such major founder event after the split of human and Neanderthal/Denisovan ancestors or a sudden change in morphology at this critical period of time has been lacking [59,60].

#### The need for whole mtDNA sequences

Two major limitations of the RFLP approach and D-loop sequencing were the small number of bases and therefore limited molecular resolution for distinguishing variation at sub-regional level, and, secondly, low robustness of the phylogenetic inferences caused by the high mutation rate of the hypervariable regions. Hypervariable positions are known to undergo multiple parallel mutations in many lineages and this parallelism becomes a significant confounding factor even within a short time scale of few tens of thousands of years of evolutionary history. These recurrent mutations generate phylogenetic uncertainty, also known as homoplasy, which even in case of the presence of only a few tens of such sites and sample size of few tens of individuals can lead to the problem of millions of trees having equal length or likelihood to be consistent with the data. Network approaches [61] were developed to visualize the complexity of parallel relationships among the mitochondrial lineages but for solving them more data from the conservative regions of mtDNA were required. Further improvements of the classical Sanger sequencing technology in the end of the last century enabled the sequencing of the whole mtDNA for the purpose of human evolutionary studies. Progress in the technology use was significantly motivated by our need to understand the genetics of disease.

**Figure 1:**
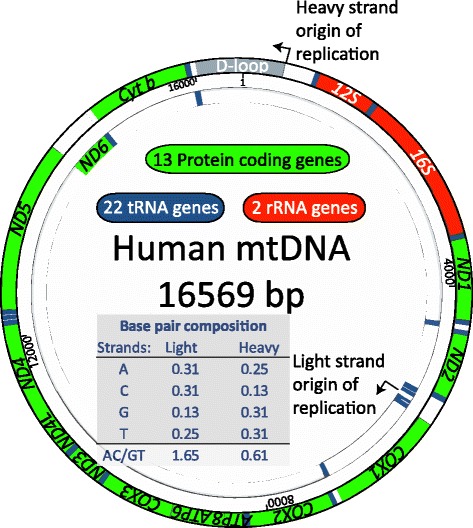
**Functional map of mtDNA.** As in other vertebrates, human mtDNA is circular and characterized by high gene density and strand asymmetry. The heavy strand encodes all mtDNA genes except for the ND6 and has a high GT/AC ratio. Protein coding, rRNA and tRNA genes are shown in boxes distinguished by different colours. Adapted from Schon *et al*. [63].

When deleterious mutations occur over time natural selection prohibits them reaching high frequency and removes them from circulation. One of the key drivers of the study of full mtDNA sequences has been medical genetics and, in particular, the need to understand the genetic basis of mitochondrial disorders and deleterious mutations. Compared to our nuclear genes, those residing in mitochondria do not have introns and much non-coding sequence around them - the whole mitochondrial genome is densely (93%) packed with protein coding, ribosomal and transport RNA genes (Figure 1). A large proportion of positions in these genes are known to be highly conserved across different species, implying strong purifying selection, and invariable in large human cohorts likely because of being fatally deleterious or associated with disease (see MITOMAP [62]). All mitochondrial genes are viably important and diseases associated with impaired function of mitochondrial protein coding genes affect primarily muscular and neural function (for review, see [63]). Therefore, unsurprisingly, the first studies to employ the whole mtDNA sequencing approach were those attempting to uncover the causative mutations of neurodegenerative diseases [64-66].

Besides the motivation for disease studies the sequencing of whole mtDNA provided also the means for getting statistically better supported phylogenetic trees to study the history of human populations. The first worldwide survey of mtDNA whole genome sequences [38] showed with a robust bootstrap support of the internal branches that the root of the human mtDNA variation lies in Africa with TMRCA date of 171,500 ± 50,000 years and that the age of the youngest clade with African and non-African sequences was 52,000 ± 27,500 years. Other whole mtDNA studies, for example [26,45,56,67-69], based on global sampling have generally agreed with these structural findings and revealed more details of the regional patterns of diversity, time scale of the accumulation of diversity, and the female effective population size changes over time. It should be noted, though, before exploring the geographic distribution of its variation that mtDNA molecule, however well resolved its phylogeny and no matter how large the sample size, remains to be just one single genetic locus which is subject to large stochastic variation and that population level inferences of demographic history require the synthesis of evidence from many loci.

### Distribution of variation in mtDNA genomes among human populations

**Figure 2:**
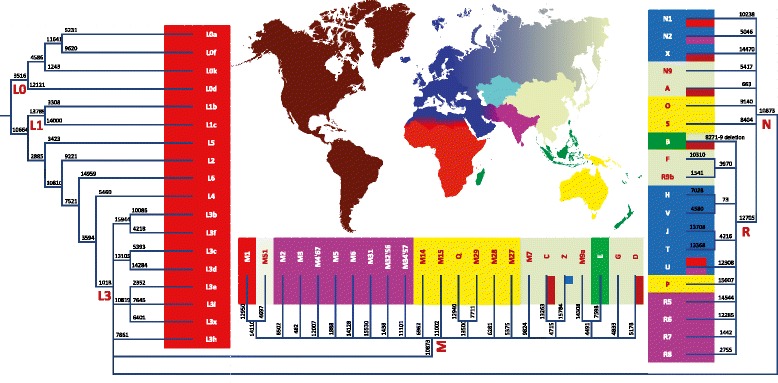
**mtDNA haplogroup tree and distribution map.** Haplogroup labels are reported according to the http://www.phylotree.org/ nomenclature [14]. Only a single branch defining marker, preferably from the coding region, is shown. The main geographic features of haplogroup distribution are highlighted with colour.

Compared to the estimates based on autosomal data the observed differences in mitochondrial sequences among human populations on a global scale are significantly higher and second only to the differences based on Y chromosomes, with Africa showing the highest within region diversity and Native Americans having the lowest [56]. As it has been repeatedly shown with ever increasing sample sizes that are reaching tens of thousands of individuals now [68], the root of the mtDNA phylogeny and the most diverse branches are restricted to African populations (Figure 2). Using the maximum molecular resolution enabled by the analysis of whole mtDNA genomes, the first seven bifurcations in this tree, in fact, define the distinction of strictly sub-Saharan African branches (L0-L6) from those that are shared by Africans and non-African populations. Analyses of whole mtDNA sequences of sub-Saharan Africans have revealed early, ca 90 to 150 thousand years (ky) old divergence of the L0d and L0k lineages that are specific to the Khoisan populations from South Africa and it has been estimated that during this time period at least six additional lineages existed in Africa with living descendants [53,54]. In contrast to the overall high basal clade diversity and geographic structure some terminal branches from haplogroups L0a, L1c, L2a, and L3e show recent coalescent times and wide geographical distribution in Africa, likely due to the recent Bantu expansion [70-72]. Given the complexity of admixture of the Bantu-speaking populations the use of whole mtDNA sequences in these studies have been instrumental in revealing the distinct autochthonous sources and ancient substructure at the background of the overall high genetic homogeneity of the Bantu speakers [70]. Outside Africa, haplogroup L0-L6 lineages are extremely rare and restricted to geographic areas that have received historic gene flow from Africa, such as Mediterranean Europe, West Asia, and Americas. On the basis of analyses of high resolution whole mtDNA sequences it has been estimated that approximately two thirds of the rare African L lineages that are found at combined frequency of <1% in Europe were brought in from Africa during the Roman times, Arab conquests and Atlantic slave trade while just one third are more likely to have been introduced earlier during pre-historic times [73].

The fact that virtually every non-African mtDNA lineage derives from just one of the two sub-clades of the African haplogroup L3 (Figure 2) has been interpreted as an evidence of a major bottleneck of mtDNA diversity at the onset of the out of Africa dispersal [74]. The magnitude of this bottleneck has been estimated from the whole mtDNA sequence data yielding the estimates of the effective population size which range between several hundred [75] and only few tens of females [56]. The separation of these two sub-clades, M and N, from their African sister-clades in L3 can be dated back to 62 to 95 kya [48] whereas the internal coalescent time estimates of the M and N founders have been estimated in the range of 40 to 70 ky [26,28,75] and suggest that their dispersal occurred probably after rather than before the eruption of Mount Toba 74 kya in Indonesia, one of the Earth’s largest known volcanic events in human history. Archaeological evidence from Jurreru River valley, India, has shown the presence of artefacts right above and below the layers of ash associated with the Toba eruption [76]. It is not clear whether the makers of these artefacts were archaic or anatomically modern humans. As in case of the global TMRCA estimate considered above the wide error ranges around the age estimates of haplogroups M and N reflect primarily the uncertainties of the mutation rate - in relative terms, the age estimates of M and N, as determined from whole mtDNA sequences form approximately one third of the total depth of the global mtDNA tree. Claims for relatively recent, post-Toba, time depth of the non-African founder-haplogroups have been recently supported by the aDNA evidence of the 45 kya Ust-Ishim skeleton whose whole mtDNA sequence falls at the root of haplogroup R [50]. While haplogroups M and N are widely spread in Asia, Australia, Oceania and Americas, the geographic distribution of each of their sub-clades has more specific regional configuration (Figure 2).

In Eurasia haplogroups U, HV, JT, N1, N2 and X are today common in Europe, Southwest Asia and North Africa [77]; haplogroups R5-R8, M2-M6 and M4’67 are restricted to South Asia [78], while haplogroups A-G, Z and M7-M9 are widespread in East Asia [79] (Figure 2). Despite the clear and distinct geographic spread patterns in extant populations it is not simple and straightforward to make inferences about the origin of these patterns and to associate the haplogroup labels with specific prehistoric events or time periods. Phylogeographic inferences made from extant variation both at low and high molecular resolution have suggested that majority of the haplogroups that are common today throughout Europe derive from the Late Glacial re-colonization event [77]. ADNA evidence, however, shows [80] that only a subset of haplogroup U variation is likely to have ancestry in pre-Neolithic Europe while other haplogroups are likely to be related with more recent episodes of gene flow and demographic events which, apparently, have quite dramatically changed the genetic landscape of the region in the past 10,000 years. ADNA analyses of the nuclear genomes of Mesolithic and Neolithic samples from Europe have suggested that the discontinuity observed in central European mtDNA types may be echoed by the appearance approximately 4,500 years ago in Europe of an ancient Near Eastern component in the autosomal genes [81].

MtDNA variation in Native Americans variation primarily falls to haplogroups A to D; X and that with the exclusion of X form a subset of the East Asian diversity [10]. Since the initial attempts to define the number of Native American founder lineages within these five basic haplogroups at low resolution attainable with RFLP and hypervariable region sequencing approaches [10,21], at least 16 sub-clades have been assigned now the founder status on the basis of whole mtDNA genome sequence analysis [82-87]. The spread of these sub-clades in North and South America has been associated with at least three distinct demographic events: (1) the main wave of the spread of the ancestors of both North and South American native populations 15–18 kya involving nine Pan-American founders A2*, B2*, C1b, C1c, C1d*, C1d1, D1, D4h3a, and D4e1c, followed potentially approximately at the same time by an inland route dispersal of C4c, X2a and X2g carriers to the east coast of the USA; (2) the spread of Paleo-Eskimo D2a [88] lineages ca 5 kya along the Arctic through northern Canada and Greenland, which were replaced, in the same region, by (3) the spread of Neo-Eskimos carrying A2a, A2b, and D3 lineages. Phylogeographic inferences from modern whole mtDNA sequence data associating the spread of haplogroup A2a lineages with Paleo-Eskimos [83] have not been supported by aDNA evidence which instead points to all available skeletal evidence that is associated with the Paleo-Eskimo cultures Saqqaq and Dorset having unusually low mtDNA diversity restricted only to haplogroup D2a [89].

The whole mtDNA sequencing of Oceanians has revealed a number of distinct mtDNA lineages that were undistinguishable at lower resolution from those spread in Mainland Asia. The peopling of Oceania has been modelled to involve at least two major demographic events: firstly, the initial settlement of Sahul (Papua New Guinea and Australia) by anatomically modern humans explains the presence of mtDNA haplogroups M14-M15, M27-M29, Q, P, O, and S only in Australia and Melanesia; secondly, this was followed by a more recent Holocene dispersal of the populations speaking Austronesian languages who would have extended widely the geographic distribution of haplogroup B4a1a1 lineages [90]. Although the high frequency of an intergenic 9-bp deletion together with a specific D-loop motif, that is characteristic to the haplogroup B4a1a1 mtDNA molecules of all Austronesian speaking populations, was noticed already in the low resolution studies of 1990’s, the employment of whole mtDNA sequencing, in combination with aDNA evidence, has made it possible now to narrow substantially down the geographic regions in Island Southeast Asia that carried the sequences directly ancestral to those of the majority of Austronesians [91-94].

### The future of whole mtDNA analyses in the era of next generation sequencing of whole nuclear genomes

Now that tens of thousands of whole mitochondrial genome sequences are already publicly available and cover virtually all extant population of the world, is there still a need for more mtDNA data and room for novel findings? Whole mitochondrial sequencing certainly continues to have an important role in forensics, in medical genetics and in ancestry and genealogy related applications because of the specific needs for mtDNA evidence in these fields. Although questions about demographic history of populations, natural selection, the extent of admixture and many other relevant aspects of genetic research of human populations can now be addressed at the level of whole genome sequences, mtDNA has continued to play an important role in the evolutionary genetic studies. MtDNA sequence variation is used in aDNA studies for the estimation of contamination levels (for example [60]) and, in turn, the accumulating aDNA evidence allows us to get increasingly more accurate insights into the complexities of mitochondrial mutation rate (Table 1). ADNA evidence combined with data from extant populations enables us, as described above, to better understand the temporal dynamics of the change of genetic diversity in regions such as Europe [80,81].

Whole mtDNA sequencing will continue to inform us about the sex specific patterns of human migrations and admixture. Consistent with the evidence from nuclear genetic loci and historical records whole mtDNA sequences of the Siddis from India have been shown to include a substantial proportion of lineages that have the closest affinity with those of the Bantu speaking populations of East Africa [95]. Because this admixture dates back only a couple of centuries it is not surprising that both sex specific loci and autosomes show consistent patterns. In contrast, other South Asian populations, such as Santhals and Mundas who speak Austroasiatic languages, have maintained the evidence of their admixed origins and Southeast Asian descent only in their Y chromosome while their mtDNA lineages cluster most closely with neighboring Indian populations [96].

The inferences of long-term effective population size from whole mtDNA and Y chromosome sequence data are continuing to provide new insights into the social behaviour of the past populations. The comparisons of female (*N*_*f*_) and male (*N*_*m*_) effective population size estimates suggest that *N*_*f*_*/N*_*m*_ ratio has been higher than 1 over the course of our evolutionary history and showing an increase in more recent times [56]. Several factors can explain *N*_*f*_*/N*_*m*_ deviations from 1, including selection, mobility and residence patterns. Analyses of populations from the Indonesian archipelago have shown that during the historic times the contacts with foreigners, such as Chinese, Indians, Arabs and Europeans, have left a noticeable imprint in the Y-chromosome variation of these indigenous populations whereas these patterns are not reflected in their mtDNA data. Whole mtDNA sequence data, on the other hand, have retained more clearly the evidence of a major geographic expansion of specific founder types, suggesting that in pre-historic times the women were more mobile than men in spreading their mitochondria from island to island [97]. This together with the findings of sex specific patterns of the Asian versus Papuan ancestry components suggests that the predominant residence pattern of the proto-Oceanic speaking populations who spread the Austronesian languages in the Pacific may have been matrilocal [90,92,98-100]. Matrilocal residence in today’s world is rare and restricted to a small number of populations, some of which have been studied to explore the effect of residence patterns on our genetic diversity [101]. Due to prevailing patrilocality the genetic differences among population are typically higher for Y chromosome than for mtDNA, although this effect has been mostly noticed at local rather than global scale [102]. It has been shown that it is crucial to use the full power of whole mtDNA sequences to reveal such differences [103].

## Conclusions

In sum, mtDNA evidence will probably continue to be important for various facets of population genetic research in the coming decades. Because of its high copy number, it will be used routinely in aDNA studies for the preliminary assessment of the quality of DNA preservation and for the evaluation of contamination. And, because of its maternal inheritance, it will continue to be informative tool for the study of sex-specific patterns in and among human populations.

## Abbreviations

aDNA: ancient DNA
HVS: hypervariable segment
mtDNA: mitochondrial DNA
*N*_f_: female effective population size
*N*_m_: male effective population size
RFLP: restriction fragment length polymorphisms
TMRCA: the most recent common ancestor
